# Evaluation of Deformation and Antibacterial Properties of Dental Alginates Mixed with Silver Nanoparticles

**DOI:** 10.3390/ma18092069

**Published:** 2025-04-30

**Authors:** Mario A. Rivera-Cortés, Nereyda Niño-Martínez, Facundo Ruiz, Brianda Karina Félix-Sicairos, Gabriel-Alejandro Martínez-Castañón

**Affiliations:** 1 Doctorado Institucional en Ingeniería y Ciencia de los Materiales, Universidad Autónoma de San Luis Potosí, Sierra Leona No. 550, Col. Lomas 2da. Sección, San Luis Potosí C.P. 78210, Mexico; m_rivec@hotmail.com; 2 Facultad de Ciencias, Universidad Autonoma de San Luis Potosí, Av. Parque Chapultepec No. 1570, Privadas del Pedregal, San Luis Potosí C.P. 78295, Mexico; facundo@fciencias.uaslp.mx; 3 Doctorado en Ciencias Biomédicas Básicas, Facultad de Medicina, Universidad Autonoma de San Luis Potosí, Av. Sierra Leona No. 550, Col. Lomas 2da. Seccion, San Luis Potosí C.P. 78210, Mexico; dra.briandafelix@gmail.com; 4 Facultad de Estomatología, Universidad Autonoma de San Luis Potosí, Av. Dr. Manuel Nava No. 2, Zona Universitaria, San Luis Potosí C.P. 78290, Mexico

**Keywords:** biomaterials, alginate, silver nanoparticles, deformation and antimicrobial activity

## Abstract

This study aimed to evaluate the effect of the incorporation of silver nanoparticles (AgNPs) of 5.57 nm size into dental alginates on their deformation and antimicrobial properties. Six experimental groups were prepared: 2 different alginates with 0.25 wt% AgNPs, 2 different alginates with 0.5 wt% AgNPs (5.57 nm), and 2 unmodified control alginate groups. The presence of AgNPs was confirmed using X-ray diffraction analysis with a Bruker D8 Advance diffractometer. Antimicrobial activity was evaluated using the Kirby-Bauer disk diffusion method (direct contact) against *E. coli* and *S. aureus* cultures incubated on Mueller–Hinton (M-H) agar at 37 °C for 24 h. The results demonstrated that the addition of 0.25% and 0.5% AgNPs significantly enhanced the antimicrobial properties of alginate (*p* < 0.05), showing clear inhibition zones against the tested microorganisms. In terms of mechanical properties, AgNPs-modified samples exhibited improved elastic recovery compared to the control group (*p* > 0.05). These findings suggest that incorporating silver nanoparticles into alginates could enhance their antimicrobial properties without compromising the mechanical integrity required for dental applications.

## 1. Introduction

Dental alginates were developed almost a century ago; they are still under continuous evolution and are used regularly in most dental offices to make diagnostic and digital models [1]. Alginate is characterized by its low cost in the market, and it behaves better when compared to other impression materials [2]. The impression obtained with alginates at the first dental consultation is important because it generates the initial evaluation of the patient’s oral health status [3]. Alginates for dental impressions are salts of alginic acid, a polysaccharide extracted from the cell walls of algae. Alginic acid is then converted into a salt (alginate) of sodium, potassium, or magnesium. Although alginate is insoluble in water, its alkaline salts are soluble in water, and therefore, it is used in dentistry [1,3]. Cross-infection is one of the main concerns when using alginates to produce stone models; the aim of this work was to determine whether the addition of silver nanoparticles to alginates could provide them with antibacterial properties without affecting their deformation. We did not consider the complete characterization of the alginates because the properties of these materials have already been reported elsewhere. For ALGINoplast^®^, their dynamic viscosity is reported as 90 Pa·s, the gelation time is 128.5 s, and the compressive strength of 0.82 MPa, and the gelation time for Cavex Cream is 126.5 s [4,5].

The oral cavity contains more than 1591 microbial species, including bacteria, fungi, archaea, viruses, and protozoa, colonizing the hard and soft surfaces of the mouth cavity, among which dental organs and adjacent tissues are found [6,7]. Inadequate disinfection and the lack of correct implementation of protocols result in an unsafe environment. *Staphylococcus aureus*, *Micrococcus*, *Pseudomonas*, *Bacillus*, *Acinetobacter*, *Streptococcus*, and *Candida albicans* are common in patients with prosthetic dental appliances or orthodontic appliances [8,9]. Pathogenic microorganisms cannot only be accommodated on the surface of the material but also within it, due to the hydrophilic nature of the alginate. Currently, research has shown that many impressions have been sent to laboratories without being subjected to any type of disinfection [10,11]. In addition, blood and saliva from the mouth are common oral fluids that contaminate materials [12]. The procedure of rinsing dental impressions with water before sending them to the laboratory removes waste but does not disinfect them, being an inadequate procedure for actual disinfections [10]. Spray and immersion are currently the most widely used methods to disinfect impressions; however, they require more time and cause negative effects related to the mechanical properties of the alginate [13,14]. Disinfectants applied after setting may not effectively penetrate all areas [15]. Dental alginate is prone to contamination due to its hydrophilic nature and porous structure that facilitate the penetration of microorganisms [16].

Silver nanoparticles are used clinically as FDA-approved antibacterial agents [17]. Silver nanoparticles (AgNPs) contain antimicrobial properties and are very applicable in dentistry involving dental prosthesis, adhesives, and implants [18]. In recent years, the incorporation of silver nanoparticles (AgNPs) into clinical dental materials has garnered increasing attention due to their well-documented antimicrobial properties. Several studies have reported that the addition of AgNPs to orthodontic adhesives, endodontic bioceramic sealers, and polymethylmethacrylate (PMMA) significantly reduces microbial load in the oral environment. This functionalization not only enhances the clinical performance of the materials but also improves the therapeutic efficacy of the treatments in which they are applied, favoring a better prognosis and increased longevity of dental procedures. The use of this nanotechnology represents a promising advancement toward optimizing biosafety in contemporary dental practice [19,20,21]. Its small size, large surface area, and unique physicochemical properties make it effective against a broad spectrum of bacteria, viruses, and fungi [18]. Thus, the aim of this work is to evaluate the deformation and antimicrobial properties of two commercial alginates mixed with silver nanoparticles (5.57 nm). The hypothesis was that incorporation of silver nanoparticles into dental alginates would provide antimicrobial activity to the materials without significantly affecting their mechanical performance.

## 2. Materials and Methods

### 2.1. Materials

For this in vitro study, six groups were formed, each consisting of ten samples (*n* = 10). Two commercial brands of alginate were compared: Cavex Cream © (Cavex Holland BV, Haarlem, The Netherlands) and ALGINoplast^®^ (Heraeus Kulzer, Hanau, Germany). The groups were organized as follows:

Group 1: ALGINoplast + distilled water without nanoparticles.

Group 2: ALGINoplast + distilled water and AgNPs 0.25 wt%.

Group 3: ALGINoplast + distilled water and AgNPs 0.5 wt%.

Group 4: Cavex cream + distilled water without nanoparticles.

Group 5: Cavex cream + distilled water and AgNPs 0.25 wt%.

Group 6: Cavex cream + distilled water and AgNPs 0.5 wt%.

### 2.2. Silver Nanoparticles and Preparation of Samples

The AgNPs used in this work were prepared using a chemical precipitation method with gallic acid as reducing and stabilizing agent; AgNPs showed a spherical morphology with a diameter of 5.57 nm (measured with TEM). DLS analysis showed a hydrodynamic diameter of 5.6 nm with a surface plasmon resonance maximum at 405 nm, confirming their identity. A zeta potential of −36 mV was found with a polydispersity index of 24.36%; this characterization is presented in full by Navarrete-Olvera et al. [19].

For the control groups (1 and 4), pure distilled water was used for alginate preparation without any additives. For 0.25 wt% AgNPs groups (2 and 5), 2.3 g of alginate powder was combined with 4.3 mL distilled water and 0.625 mL AgNPs (1070 ppm). For 0.50 wt% AgNPs groups (3 and 6), 2.3 g of alginate powder was mixed with 3.75 mL distilled water and 1.25 mL of AgNPs (1070 ppm). Both Cavex cream and ALGINoplast^®^ require immediate pouring to maintain their dimensional stability [22].

### 2.3. Measurement of Deformation

The preparation of test and control samples was conducted following the protocol outlined in the ADA specification no. 18 [23] and ISO: 21563:2021 [24] using a master die. The specimens were created by placing a ring (3 cm inside diameter, 16 mm high) on a flat glass plate and filling the ring slightly more than halfway with either dental alginate (control group) or modified (test group).

A metal mold (12.7 mm inside diameter, 25.4 mm outside diameter, 19.2 mm high) was then placed inside the ring and pressed into the material until it contacted the glass plate, allowing the excess material to exude to the top mold. A solid brass plate was positioned on the upper surface of the ring to remove any surplus material. The prepared specimen was transferred to a deformation apparatus, where a lightweight plate (15 × 15 mm in size and 2 mm thick) was placed on the specimen. The foot of a dial indicator was then brought into contact with the plate to measure deformation [25].

The test was carried out in accordance with the following time schedule, where t was the initial setting time: (a) t + 45 s—the spindle of the dial indicator was lowered so that it came into contact with the plate on the specimen; (b) t + 55 s—the dial indicator was read, the value was recorded as the initial reading, and the spindle was fixed in the “up” position; (c) t + 60 s—the specimen was deformed to a height of 16 mm ± 0.1 mm within 1 s, and this deformation was maintained for 5 s ± 0.5 s, then released; (d) t + 90 s—the spindle of the dial indicator was lowered so that it came into contact with the plate on the specimen; (e) t + 100 s the dial indicator was read, and the value was recorded.

The elastic recovery was calculated as a percentage using the following formula from ANSI/ADA specification no. 18 for irreversible hydrocolloid impression material [23,25]:

(1) $$\left(1-\frac{a-b}{16}\right)$$

where the constant 16 was the length of the mold in millimeters. All tests were designed in accordance with ANSI/ADA specification no. 18 for irreversible hydrocolloid impression material [23,25].

The deformation specimens were prepared in a similar manner to the other specimens, and each section was placed on the metal slab of the Universal Testing Machine (Multitest 1-d, Mecmesin, Horsham, UK) and tested for compressive strength. The specimens were loaded continuously and as uniformly as possible to produce an average rate of 100 N/min ± 20 N/min until the point of yield.

### 2.4. Surface Roughness Analysis

Measurements for surface roughness were obtained from three different points in the samples using atomic force microscopy (AFM) (Nanosurf Easy Scan 2, SPM Electronics, Liestal, Switzerland) in contact mode with a silicon nitride (SiN) scanning rate of 49.5 μm/s. The values used for the short cantilever were spring constant 0.1 N/m, resonant frequency 28 kHz, length 225 μm, mean width 28 μm, thickness 1 μm, tip height 14 μm, and radius <10 nm. A calibration grid of silicon oxide on a silicon substrate (Nanosurf AG, CH-4410, SPM Electronics, Liestal, Switzerland) with XY periodicity of 10 μm and a Z height of 119 nm was used to calibrate the instrument before the evaluation. The Nanosurf Easy Scan 2 software (version 1.6) was used to measure the AFM parameters. The surface roughness was quantified using roughness average (Sa), which represents the arithmetical mean of the absolute values of the scanned surface profile.

### 2.5. Scanning Electron Microscopy

Samples with and without silver nanoparticles were imaged using scanning electron microscopy. Disks of each sample were prepared and allowed to dry under room conditions; after that, samples were carbon-coated and analyzed using a JEOL JSM-6610 (JEOL Ltd., Tokyo, Japan) at 25 kV. Chemical analysis was performed using an EDS analyzer attached to the microscope.

### 2.6. Measurement of Antibacterial Effect

Alginate disks impregnated with AgNPs (0.25 and 0.5 wt%) were prepared to assess their antimicrobial activity. The samples were standardized in terms of dimensions, with a diameter of 10 mm and a thickness of 2 mm. Six experimental groups were established, each consisting of 10 discs, resulting in a total of 120 specimens. Each group was inoculated with specific bacterial strains to evaluate the interaction between the alginate discs with AgNPs and the selected bacteria. The disks were placed on Mueller–Hinton (M-H) agar plates inoculated with lawn cultures of *Escherichia coli* (ATCC 25922) and *Staphylococcus aureus* (ATCC 25923) and incubated at 37 °C for 24 h [24]. The bacterial inoculum was prepared using the McFarland standard (0.5), corresponding to a concentration of 10^8^ CFU/mL. After 24 h of incubation, the diameter of the growth inhibition zones was measured in millimeters.

### 2.7. X-Ray Diffraction

X-ray diffraction has now become a standard technique for investigating and analyzing the properties of crystalline materials. Its non-destructive nature allows for the preservation of sample integrity, making it suitable for a wide range of scientific and industrial applications. Through XRD, a deeper understanding of material properties can be achieved, which is essential for advancing technological innovations and improving material performance in various applications [26]. The results obtained from the diffractograms indicate the presence or absence of specific materials [27].

To corroborate the presence of silver nanoparticles in the alginate, the X-ray diffraction test was carried out, which consists of analyzing the composition of a material and its components, in this case, the alginate; for the analysis of the samples, three different mixtures previously crushed in solid form were used until they were turned into powder; the specimens were divided into three for each test: specimens mixed with alginate and silver nanoparticles 0.25 wt% (0.25 wt% group), specimens mixed with alginate and silver nanoparticles 0.5 wt% (0.5 wt% group), and specimens mixed with distilled water and alginate (control group).

The analysis was performed using an X-ray diffractometer (Bruker model D8 Advance Da Vinci made in Karlsruhe, Germany) with the following diffracted conditions: 2theta = 4–90°, step size 0.02°, time/step= 0.3 seg, with a time of diffracted of 21 min and the Haz generation = 40 kV 35 mA.

### 2.8. Statistical Analysis

Statistical analysis comprised both descriptive and analytical components. The descriptive phase evaluated deformation measurements, antimicrobial activity, detailed reproduction, and other relevant characteristics of the control and test specimens. Data were analyzed using 1-way ANOVA, and significant differences between the control and test groups were compared with the Dunnett test (*p* > 0.05). Statistical analysis was performed using GraphPad Prism 9 version 9.0.2 (134) for MacOs.

## 3. Results

### 3.1. Measurement of Deformation

Table 1 presents the recovery from deformation, expressed as a percentage, for dental alginates incorporated with different concentrations of silver nanoparticles. Two distinct dental alginates were evaluated: Cavex Cream (© Cavex Holland BV, Haarlem, The Netherlands) and ALGINoplast^®^ (Heraeus Kulzer, Hanau, Germany).

In the control group, both Cavex and ALGINoplast^®^ demonstrated high recovery percentages, with Cavex achieving 97.43 ± 0.59% and ALGINoplast 98.47 ± 0.31%. Upon the incorporation of silver nanoparticles (AgNPs) at concentrations of 0.25 wt% and 0.50 wt%, slight variations in recovery percentages were observed. For Cavex, the recovery percentages were 97.36 ± 0.42% (*p* = 0.9246) and 97.41 ± 0.48% (*p* = 0.9910), at the respective concentrations. Similarly, ALGINoplast exhibited recovery percentages of 98.21 ± 0.40% (*p* = 0.1851) and 98.20 ± 0.30% (*p* = 0.1591) under the same concentrations. These findings indicate that the addition of AgNPs did not significantly affect the elastic recovery properties of the tested alginate materials.

The marginal variations observed appear to fall within the acceptable range for these materials, suggesting their potential suitability for dental applications, where recovery from deformation is a critical parameter. Further statistical analyses are warranted to provide deeper insights into the significance of these differences.

### 3.2. Surface Roughness

The surface roughness values of the alginates without AgNPs are 1360 nm for Cavex and 1417 nm for ALGINoplast; when silver nanoparticles were added to the alginates, there were an increment in the roughness for both the materials and the silver concentrations but without a significant difference (*p* > 0.05) when compared with the control group for each alginate, see Table 2.

**Figure 1:**
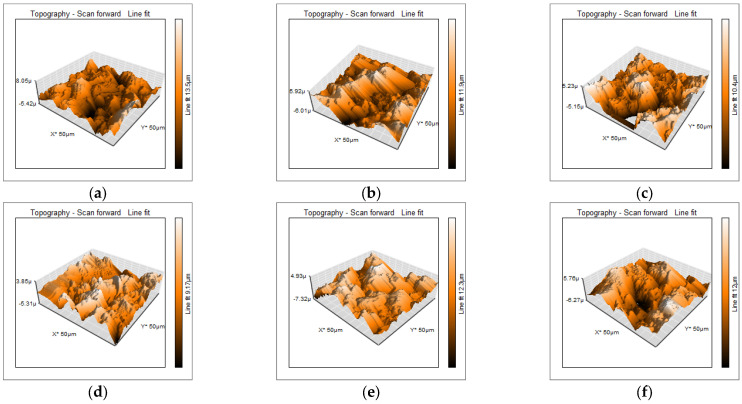
3D atomic force microscopy images of (**a**) Cavex; (**b**) Cavex with 0.25 wt% AgNPs; (**c**) Cavex with 0.5 wt% AgNPs; (**d**) ALGINoplast; (**e**) ALGINoplast with 0.25 wt% AgNPs; and (**f**) ALGINoplast with 0.5 wt% AgNPs.

Representative 3D atomic force microscopy images of each group are presented in Figure 1; as we can see, samples are homogeneous in their surface with no visible alteration due to the presence of silver nanoparticles.

### 3.3. Scanning Electron Microscope Analysis

**Figure 2:**
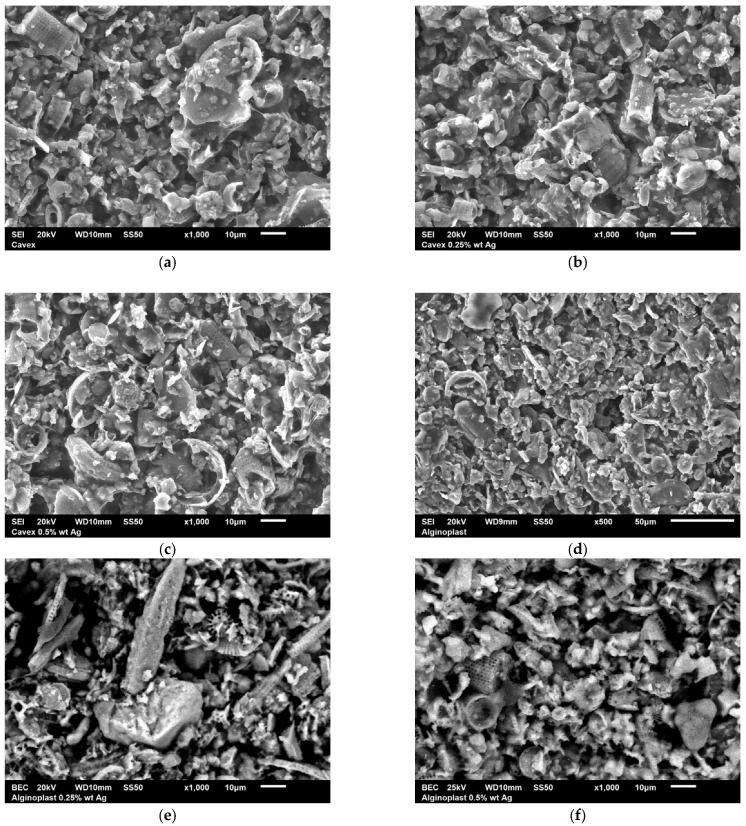
SEM images of (**a**) Cavex; (**b**) Cavex with 0.25 wt% AgNPs; (**c**) Cavex with 0.5 wt% AgNPs; (**d**) ALGINoplast; (**e**) ALGINoplast with 0.25 wt% AgNPs; and (**f**) ALGINoplast with 0.5 wt% AgNPs.

SEM images of the samples with and without silver nanoparticles are presented in Figure 2. The inorganic phase of the materials can be observed with different sizes and shapes; most of the particles are irregular, but we also found cylindrical and spherical particles with perforations along their body; these are typical morphologies for filler in dental alginates. The presence of silver nanoparticles could not be observed, but the chemical analysis demonstrated the presence of silver nanoparticles (Figure S1).

### 3.4. Antimicrobial Activity

Table 3 presents the antimicrobial activity of dental alginates incorporated with silver nanoparticles, expressed in mm. The data are reported as mean values accompanied by their respective standard deviations.

The mechanisms of action for solid dental materials could be classified into two categories: antimicrobial-releasing and contact-killing methods (potentiated surfaces and substances that do not allow for bacterial adhesion). In this work, control groups presented only contact inhibition activity with no detectable halo beyond the limits of the disks. Upon incorporating silver nanoparticles at concentrations of 0.25 wt% and 0.50 wt%, there was a discernible increase in antimicrobial activity for both ALGINoplast^®^ and Cavex. For ALGINoplast^®^ against *E. coli*, the mean antimicrobial activity reached 10.52 ± 0.17 (*p* < 0.05) and 10.54 ± 0.24 (*p* < 0.05) mm at 0.25 wt% and 0.50 wt%, respectively. Similar trends were observed against *S. aureus*, with mean values of 10.61 ± 0.20 (*p* < 0.05) and 10.63 ± 0.31 (*p* < 0.05) mm. Cavex group exhibited antimicrobial activity with mean values of 10.03 ± 0.087 and 10.21 ± 0.08 (*p* < 0.05) mm against *E. coli* and 10.31 ± 0.14 (*p* < 0.05) and 10.35 ± 0.19 (*p* < 0.05) mm against *S. aureus* at 0.25 wt% and 0.50 wt%, respectively.

**Figure 3:**
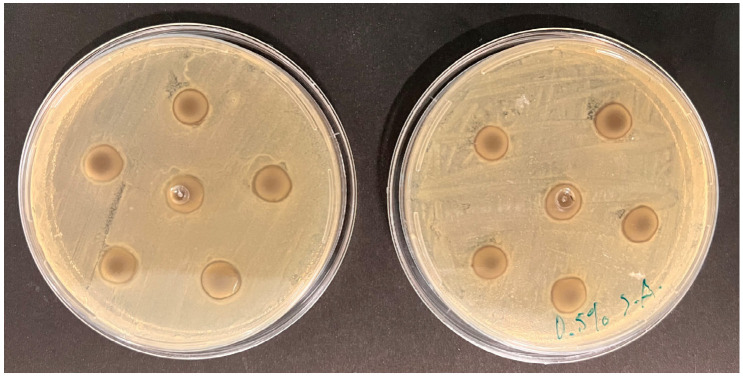
Measurement of the antibacterial effect of the materials tested.

These results suggest that the incorporation of silver nanoparticles enhances the antimicrobial activity of both ALGINoplast^®^ and Cavex against *E. coli* and *S. aureus*. The observed variations in antimicrobial activity can be attributed to the different concentrations of silver nanoparticles, which could diffuse through the agar and release silver ions. The inhibition zones (Figure 3) were measured in four directions, and the average values were recorded for each disk [28].

### 3.5. The X-Ray Diffraction

The diffraction test results revealed a high degree of similarity among the three samples analyzed within the 4–90° range on the diffractometer. Notably, the composition of the alginate remained unaltered across all samples.

Figure 2 presents the XRD results of the Cavex alginate mixed with silver nanoparticles at concentrations of 0% wt, 0.25% wt, and 0.5% wt. The graph highlights the compositional consistency among the samples, indicating no significant variations across the tested formulations.

The following components were detected in the composition of Calvex alginate (Figure 2a): Cristobalite, syn; Gypsum, Spencerite, Syngenite, syn; Calcium silicon oxide hydroxide hydrate, Parahopeite, and Glutaric acid. For the samples mixed with silver nanoparticles (Figure 2b,c), the components are the same with the addition of silver.

**Figure 4:**
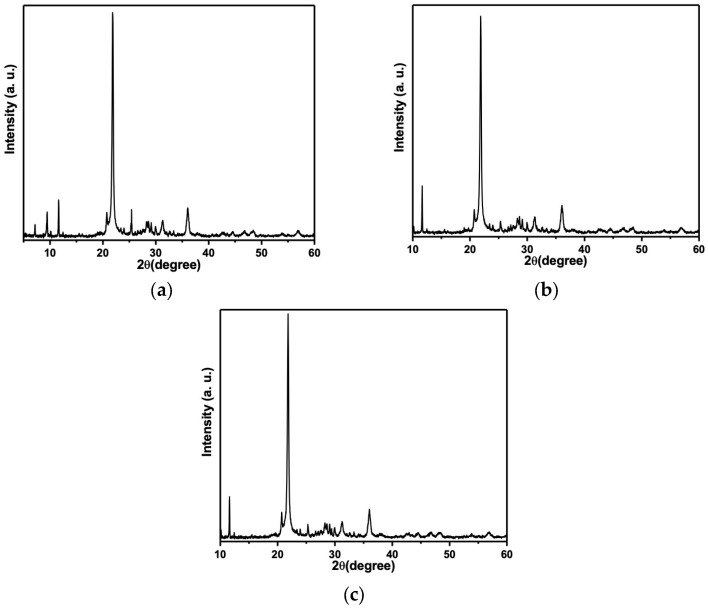
(**a**) Diffraction pattern of the Cavex alginate without silver nanoparticles. (**b**) Diffraction pattern of the Cavex alginate mixed with silver nanoparticles at 0.25%. (**c**) Diffraction pattern of the Cavex alginate mixed with silver nanoparticles at 0.5%.

The X-ray diffraction test made to alginate ALGINoplast with silver nanoparticles at 0.25% and 0.5% (Figure 3 and Figure 4) results in the following materials in its composition: Silver, Cristobalite, Gypsum, Spencerite, Calcium silicon oxide hydroxide hydrate, Parahopeite, and Glutaric acid.

The results indicate that the silver nanoparticles did not undergo a chemical reaction when mixed with the alginate. Furthermore, the structure of the alginate remained unaltered, as evidenced by the consistent peak positions observed in the diffraction analysis, with no significant shifts detected between them.

## 4. Discussion

Saliva and blood are considered the main contaminants of dental alginate impressions taken from patients, representing a significant risk of cross-contamination and transfer of infectious microorganisms between dental health professionals, including operators, assistants, and dental laboratories. Several studies have documented the presence of pathogens such as *S. mutans* and *E. coli* on plaster models, which may contribute to the occurrence of dental infections both in clinics and laboratories. This phenomenon underlines the importance of implementing proper disinfection and handling protocols for materials to minimize the risk of infection transmission [29].

The porous structure of dental alginates facilitates the retention of microorganisms both on their surface and within the material. To render a dental alginate self-disinfecting, it is impregnated with a disinfectant, enabling both internal and external disinfection. This approach overcomes the limitations of disinfectants that act exclusively on the external surface, with the objective of preventing microbial survival within the pores of the setting material [29]. It is important to produce a self-disinfectant alginate without altering other properties as the mechanical and surface properties; our results showed no alteration in the recovery and surface roughness properties of the materials tested.

In this study, 5.57 nm silver nanoparticles (AgNPs) were incorporated into dental alginates at molar concentrations of 10^−1^ M, specifically at 0.25 and 0.50% by weight. This approach builds on the work of Ginjupalli et al. 2016, who used silver nanoparticles (AgNPs) ranging in size from 80 to 100 nm at concentrations of 0.5, 1, 2, and 5% by weight [30]. The findings of these studies indicate that neither the size nor the concentration of the nanoparticles significantly affects their antibacterial properties. Even at lower AgNP concentrations, antimicrobial efficacy was maintained [31].

In contrast to the methodology employed by Omidkhoda et al. (2019), this study incorporated the direct contact method to assess antimicrobial activity [13]. This approach was specifically chosen over the disk diffusion or agar well methods due to its closer approximation to clinical conditions. The direct contact method enables us to differentiate between contact inhibition-only materials and releasing inhibition materials; our group has demonstrated that the incorporation of silver nanoparticles into dental materials such as alginates, orthodontic adhesives, and endodontic materials can improve their antibacterial activity by releasing silver nanoparticles or silver ions [19].

The degree of gelation and cross-linking in alginate plays an important role in determining its properties; the size and distribution of the incorporated nanoparticles are critical; the distribution improves the mechanical reinforcement, while agglomeration can debulk the material. Chemical interactions between particles can alter gelation, which influences the overall stability [16]. In this study, both ALGINoplast and Cavex groups did not observe any defects upon deformation; this can be attributed to the correct distribution of AgNPs in alginate. It is important to consider that humidity and temperature cannot be controlled factors in the dental clinic; however, an attempt was made to use water at 23 °C, simulating the average temperature of the dental office [32].

Like the methodology employed by Anastasiiadou et al. (1996), X-ray diffraction was utilized in this study not only to identify the components of the alginate but also to confirm the presence of silver nanoparticles (AgNPs) within the prepared formulation [27]. We can assert that the presence of silver nanoparticles is not limited to the surface of the material but is also distributed internally. This internal distribution provides a superior bactericidal effect against the bacteria, suggesting its potential as an antimicrobial agent in dental alginates [16].

These findings highlight the potential of incorporating nanomaterials into dental impression materials into dental impression materials to enhance their performance and ensure high-quality dental restorations.

Compared to the existing literature, our study represents a significant advance in the application of silver nanoparticles (AgNPs) in dental alginates. Using X-ray diffraction, we have evidenced the presence of both internal and external AgNPs in the material. More importantly, our findings indicate that, despite the gelation of the material upon mixing, this does not affect its physical and mechanical properties.

## 5. Conclusions

The incorporation of silver nanoparticles into ALGINoplast^®^ and Cavex Cream Alginate significantly enhances their antimicrobial properties, demonstrating notable effectiveness against pathogenic microorganisms such as *E. coli* and *S. aureus*. This improvement is particularly crucial for ensuring safety in dental procedures, especially for patients utilizing prosthetic devices or orthodontic appliances.

In addition, it is highlighted that these biomaterials do not alter the physical or mechanical properties of dental alginate, which makes them especially useful in dental applications. These results not only offer a potential solution to reduce cross-contamination but also open the door to future clinical trials.

## Figures and Tables

**Table 1:** Recovery from deformation, in percentage of dental alginates incorporated with silver nanoparticles.

Concentration	Cavex	*p*	ALGINoplast^®^	*p*
Control	97.43 ± 0.59	Na	98.47 ± 0.31	Na
0.25 wt%	97.36 ± 0.42	0.9246	98.21 ± 0.40	0.1851
0.50 wt%	97.41 ± 0.48	0.9910	98.20 ± 0.30	0.1591

Data are presented as number (percentage) mean ± SD and analyzed using a with 1-way ANOVA, and significant differences between the control and test groups were compared with the Dunnett test. *n* = 10 for each group. Abbreviations: wt% = weight percentage.

**Table 2:** Surface roughness (Sa, nm) of dental alginates incorporated with silver nanoparticles.

Concentration	Cavex (nm)	*p*	ALGINoplast (nm)	*p*
Control	1360 ± 279.8	-	1417 ± 251.3	-
0.25 wt%	1480 ± 201.2	0.400	1653 ± 41	0.184
0.50 wt%	1664 ± 124.5	0.100	1842 ± 117.8	0.057

Data are presented as mean ± SD and were analyzed using *t* test for the ALGINoplast group and Mann–Whitney for the Cavex group. Abbreviations: wt% = weight percentage.

**Table 3:** Antimicrobial activity, in millimeters (mm), of irreversible hydrocolloids incorporated with silver nanoparticles (mean ± SD).

	ALGINoplast^®^		Cavex			
Concentration	*E. coli*	*S. aureus*	*p*	*E. coli*	*S. aureus*	*p*
Control	10.00 ± 0.00	10.00 ± 0.00	NA	10.00 ± 0.00	10.00 ± 0.00	NA
0.25 wt%	10.52 ± 0.17	10.61 ± 0.20	<0.0001	10.03 ± 0.10	10.31 ± 0.19	<0.0001
0.50 wt%	10.54 ± 0.24	10.63 ± 0.31	<0.0001	10.21 ± 0.08	10.35 ± 0.14	<0.0001

Data are presented as number (percentage) mean ± SD and analyzed using a 1-way ANOVA, and significant differences between the control and test groups were compared with the Dunnett test. *n* = 10 for each group. Abbreviations: NA = no antibacterial activity observed; wt% = weight percentage.

## Data Availability

The data used to support the findings of this study are included within the article.

## Supplementary Materials

The following supporting information can be downloaded at: https://www.mdpi.com/article/10.3390/ma18092069/s1, Figure S1: EDS results for dental alginates with nanoparticles. (a) ALGINoplast with 0.5 wt% AgNPs; (b) ALGINoplast with 0.25 wt% AgNPs; (c) Cavex with 0.5 wt% AgNPs; (d) Cavex with 0.25 wt% AgNPs; Figure S2: X-ray diffraction test with the samples (a) ALGINoplas at 0.25% AgNPs and (b) ALGINoplas at 0.5% AgNPs.
