# Supplementary material for: Evaluation of Deformation and Antibacterial Properties of Dental Alginates Mixed with Silver Nanoparticles
Source: Materials (Basel). 2025 Apr 30;18(9):2069. doi: 10.3390/ma18092069 (PMC12072492; doi:10.3390/ma18092069)
Supplement: Supplementary file 1 [file materials-18-02069-s001.zip › materials-3566985-supplementary.pdf]

Supplementary Material

Evaluation of Deformation and Antibacterial Properties of Dental Alginates Mixed with Silver Nanoparticles

Mario A. Rivera-Cortés [1], Nereyda Niño-Martínez [2,*], Facundo Ruiz [2], Brianda Karina Félix-Sicairos [3] and Gabriel-Alejandro Martínez-Castañón [4,*]

Figure S1 shows EDS results of the alginates with silver nanoparticles, AgNPs were not visible using SEM images but it was detected with chemical analysis.

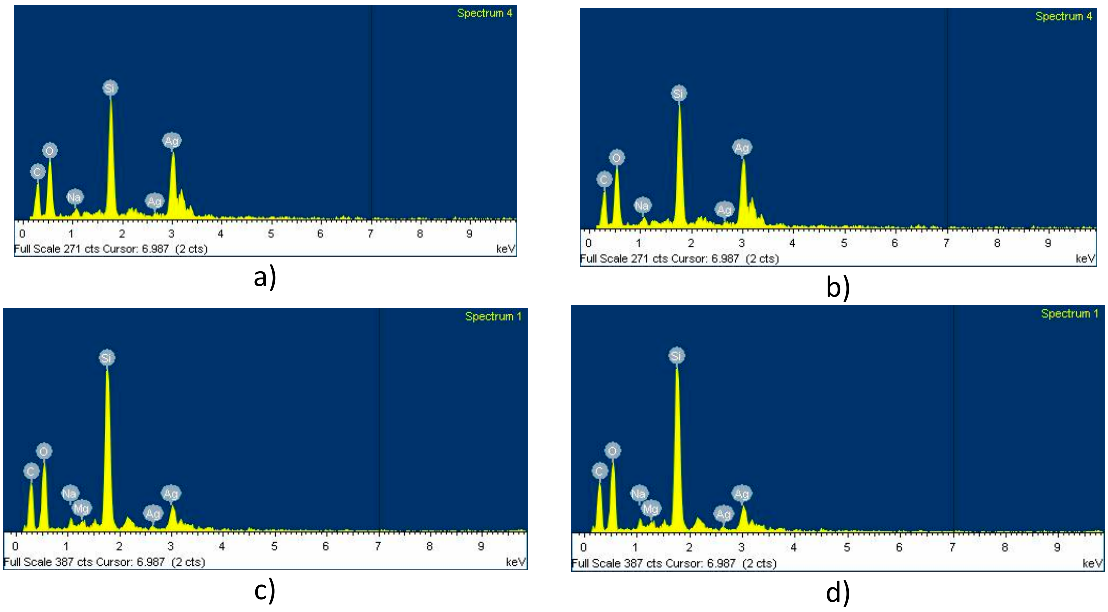

Figure S1. EDS results for dental alginates with nanoparticles. a) ALGINoplast with 0.5 wt% AgNPs; b) ALGINoplast with 0.25 wt% AgNPs; c) Cavex with 0.5 wt% AgNPs; d) Cavex with 0.25 wt% AgNPs

Figure S2 shows diffraction patterns for ALGINoplast with silver nanoparticles.

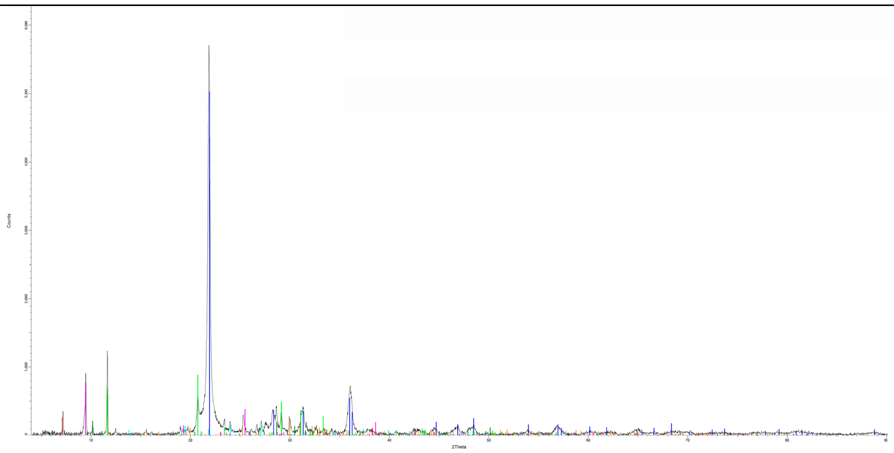

a)

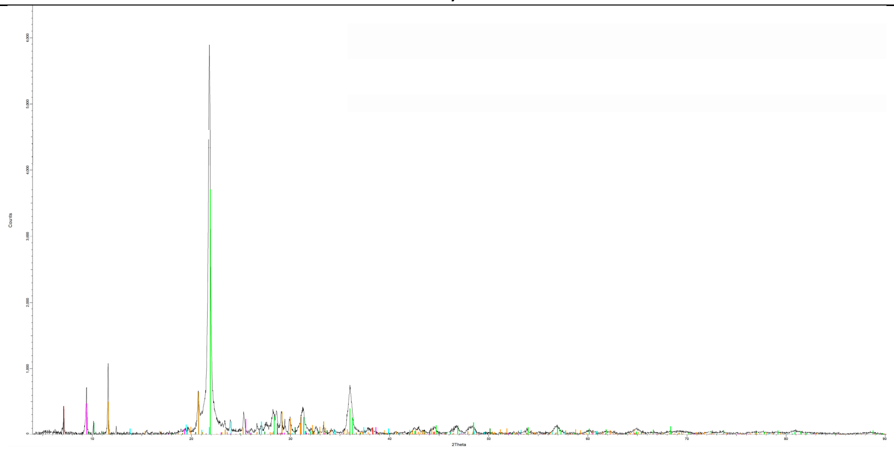

b)

Figure S2. X-ray diffraction test with the samples a) ALGINoplas at 0.25% AgNPs and b) ALGINoplas at 0.5% AgNPs.
